# Supplementary material for: Global hypo-methylation in a proportion of glioblastoma enriched for an astrocytic signature is associated with increased invasion and altered immune landscape
Source: eLife. 2022 Nov 22;11:e77335. doi: 10.7554/eLife.77335 (PMC9681209; doi:10.7554/eLife.77335)
Supplement: Figure 2—source data 1. [file elife-77335-fig2-data1.zip › Figure_2_source_data_1/Figure_2B/homerResults/motif65.similar.html]

motif65

## Information for motif65

G
A
T
C
A
C
T
G
A
C
T
G
A
C
T
G
C
T
G
A
A
C
G
T
A
C
T
G
C
T
A
G
A
C
T
G
A
C
G
T
A
T
C
G
C
G
A
T
A
C
T
G
T
G
C
A
A
C
T
G
C
A
G
T
C
T
A
G
A
C
T
G
C
G
A
T
A
C
T
G
G
A
C
T
A
G
T
C
G
C
A
T
T
G
A
C
C
A
T
G
A
C
T
G
A
C
T
G
C
G
T
A
C
A
G
T
A
C
T
G
A
C
T
G
C
T
A
G
A
G
C
T
T
C
A
G
C
G
A
T
A
C
T
G
T
C
G
A
A
C
T
G
A
G
C
T
C
T
A
G
A
T
C
G
A
G
C
T
A
C
T
G
A
C
G
T
A
G
T
C
  
Reverse Opposite:  

T
C
A
G
G
T
C
A
G
T
A
C
C
T
G
A
A
T
G
C
A
G
T
C
C
T
G
A
A
G
T
C
A
C
G
T
G
T
A
C
C
G
T
A
A
G
T
C
T
C
G
A
A
G
T
C
A
G
T
C
A
G
T
C
G
T
C
A
C
G
A
T
G
T
A
C
A
G
T
C
G
T
A
C
A
C
T
G
C
G
A
T
A
C
T
G
C
T
G
A
A
G
T
C
C
G
T
A
A
G
T
C
A
G
T
C
G
T
C
A
G
T
A
C
A
C
G
T
A
G
T
C
G
C
T
A
A
T
G
C
G
T
C
A
T
G
A
C
A
G
T
C
A
G
T
C
G
T
C
A
G
A
C
T
G
T
A
C
G
T
A
C
G
T
A
C
C
T
A
G
  

|  |  |
| --- | --- |
| p-value: | 1e-15 |
| log p-value: | -3.477e+01 |
| Information Content per bp: | 1.875 |
| Number of Target Sequences with motif | 119.0 |
| Percentage of Target Sequences with motif | 5.15% |
| Number of Background Sequences with motif | 9.0 |
| Percentage of Background Sequences with motif | 2.54% |
| Average Position of motif in Targets | 102.4 +/- 46.8bp |
| Average Position of motif in Background | 99.7 +/- 44.4bp |
| Strand Bias (log2 ratio + to - strand density) | -0.0 |
| Multiplicity (# of sites on avg that occur together) | 1.18 |
| Motif File: | file (matrix) reverse opposite |

### Similar de novo motifs found

|  |  |  |  |  |  |  |  |
| --- | --- | --- | --- | --- | --- | --- | --- |
| Rank | Match Score | Redundant Motif | P-value | log P-value | % of Targets | % of Background | Motif file |
| 1 | 0.880 | A T G C C A T G C T A G T C A G T C G A C A G T C A T G C T A G A T C G C A G T T A C G C A G T T A C G T C G A T A C G C A G T C T A G A T C G C A G T C T A G A C G T T A G C G C A T T A G C C T A G C T A G T A C G T C G A C A G T T C A G C T A G T C A G A C G T T C A G A C G T | 1e-3 | -7.497272 | 1.56% | 0.87% | motif file (matrix) |
| 2 | 0.730 | C T A G A C G T C A T G A C T G C A G T A T C G A C G T A T G C G C A T A G T C T C A G C A T G C T A G T C G A C G A T A C T G C A T G C A T G C A G T A T C G C A G T T A C G G C T A A T C G G C A T | 1e-1 | -4.541342 | 3.03% | 2.27% | motif file (matrix) |
